# Efficient Hybrid Emergency Aware MAC Protocol for Wireless Body Sensor Networks

**DOI:** 10.3390/s18103572

**Published:** 2018-10-22

**Authors:** Nadine Bou Dargham, Abdallah Makhoul, Jacques Bou Abdo, Jacques Demerjian, Christophe Guyeux

**Affiliations:** 1 Faculty of Engineering, Notre Dame University, Deir El Kamar P.O. Box 72, Lebanon; nboudargham@ndu.edu.lb; 2 Femto-ST Institute, UMR CNRS 6174, Université de Bourgogne Franche-Comté, 90000 Besançon, France; christophe.guyeux@univ-fcomte.fr; 3 Faculty of Natural and Applied Sciences, Notre Dame University, Deir El Kamar P.O. Box 72, Lebanon; jbouabdo@ndu.edu.lb; 4 LARIFA-EDST, Faculty of Sciences, Lebanese University, 90656 Fanar, Lebanon; jacques.demerjian@ul.edu.lb

**Keywords:** BSN, MAC, DTDMA, DS-CDMA, delay, packet drop, energy consumption, OPNET

## Abstract

In Body Sensor Networks (BSNs), two types of events should be addressed: periodic and emergency events. Traffic rate is usually low during periodic observation, and becomes very high upon emergency. One of the main and challenging requirements of BSNs is to design Medium Access Control (MAC) protocols that guarantee immediate and reliable transmission of data in emergency situations, while maintaining high energy efficiency in non-emergency conditions. In this paper, we propose a new emergency aware hybrid DTDMA/DS-CDMA protocol that can accommodate BSN traffic variations by addressing emergency and periodic traffic requirements. It takes advantage of the high delay efficiency of DS-CDMA in traffic burst, and the high energy efficiency of DTDMA in periodic traffic. The proposed scheme is evaluated in terms of delay, packet drop percentage, and energy consumption. Different OPNET simulations are performed for various number of nodes carrying emergency data, and for various payload sizes. The protocol performance is compared to other existing hybrid protocols. Results show that the proposed scheme outperforms the others in terms of delay and packet drop percentage for different number of nodes carrying emergency data, as well as for different payload sizes. It also offers the highest energy efficiency during periodic observation, while adjusting the energy consumption during emergency by assigning spreading codes only to nodes holding emergency data.

## 1. Introduction

Body Sensor Networks (BSNs) consist of small medical sensors implanted in or placed on the human body to capture physiological data, and send them wirelessly to health care providers to take appropriate actions accordingly [1]. BSNs have gained a lot of interest lately due to the wide medical and non-medical services they can provide, such as abnormal conditions detection, emotion detection, entertainment applications, secure authentication, etc. [2,3].

Two kinds of events are reported during patient monitoring: periodic and emergency events [4]. Emergency occurs when the monitored person’s activity or ambient environment suddenly changes. One of the most important requirements of BSNs is to handle emergency events reliably and with the lowest delay in order to take fast actions and save people’s lives [5,6].

Traffic in BSN is dynamic by nature. In general, the traffic rate is stable and relatively low in case of periodic observation (between 1–20 packets/s), and can become very high in case of emergency (between 50–100 packet/s). The main reason for this traffic burst is that physiological parameters are highly correlated, and therefore upon emergency, many sensors will need to send their data simultaneously to the coordinator at high rates to appropriately assess the patient’s situation [7,8,9]. During periodic observations (non-emergency cases), the main concern is to guarantee high energy efficiency; whereas during emergency, the main concern is to guarantee reliable and real-time transmission of critical data within strict delay requirements (less than 125 ms) [5,6].

Transmission of data with adequate QoS largely depends on the choice of the Medium Access Control (MAC) protocol. For this reason, it is essential to design dynamic MAC protocols that can adapt to BSN traffic variations, and address different traffic requirements of nodes.

In general, MAC protocols for BSN are classified as contention-based, contention-free, and hybrid [10]. Example of contention-based protocols includes Carrier Sense Multiple Access with Collision Avoidance (CSMA/CA), where nodes compete for channel sharing and transmit their data when the medium is free. Whereas Dynamic Time Division Multiple Access (DTDMA), Frequency Division Multiple Access (FDMA), and Direct Sequence Code Division Multiple Access (DS-CDMA) are examples of contention-free protocols, where nodes follow a certain schedule to transmit their data without competition. Hybrid protocols use a combination of both contention-based and contention-free schemes to satisfy certain application’s requirements.

Few protocols were suggested in literature to deal with emergency and dynamic traffic in BSN. They are mainly based on DTDMA and CSMA/CA protocols [11,12,13,14,15,16,17]. However, our previously conducted study in [18] proved that both DTDMA and CSMA/CA do not perform well in high traffic environments and induce large delays due to queuing in DTDMA and the contention nature of CSMA/CA. Therefore these two schemes are not reliable to be adopted in emergency situations where traffic rates become very high. On the other hand, this same study showed that DS-CDMA protocol is the most delay efficient among other contention-based and contention-free protocols in high traffic conditions. Thus in this paper, we propose an efficient emergency aware hybrid DTDMA/DS-CDMA MAC protocol, in which DTDMA is used to handle the periodic traffic while DS-CDMA handles the emergency traffic with spreading codes. The proposed scheme takes advantage of the low delay induced by DS-CDMA in high traffic environment, and the low energy consumption characteristic of DTDMA. The main aim of this protocol is to guarantee minimal delay and packet loss during emergency situations, while maintaining high energy efficiency during periodic observations.

The rest of the paper is divided as follows: related work is discussed in Section 2. The new emergency aware hybrid DTDMA/DS-CDMA scheme is described in Section 3. Simulation and comparison of the proposed scheme to existing MAC protocols, as well as discussion of the corresponding delay, packet loss and energy performance are presented in Section 4, while conclusion and future work are drawn in Section 5.

## 2. Related Work

Few MAC protocols found in literature are traffic adaptive and give special attention to urgent data delivery. These protocols are mainly based on a combination of a Contention Access Period (CAP) that uses CSMA/CA mechanism, and a Contention Free Period (CFP) that is based on DTDMA approach.

For instance, authors in [11] suggest a Preemptive slot allocation and Non-Preemptive transmission MAC (PNP-MAC) for Providing QoS in Body Area Networks. The PNP-MAC superframe is composed of a CAP, a beacon, a CFP formed of Data Transmit Slots (DTS) and Emergency Transmit Slot (ETS), and an Inactive Period (IP). The CAP period is used for DTS requests; the beacon is used by the coordinator for advertisement and slot allocation; the DTS are used for transmission of time critical data, the ETS used for emergency data delivery, and the IP is used for power saving as sensor nodes go to sleep. Even though this protocol assigns special slots (ETS) for emergency data to guarantee their delivery, CFP and IP have fixed duration, then the protocol does not ensure the immediate delivery of critical data since long delay will occur before finding an available emergency slot.

In [12], authors present a Low-Delay and Traffic-Adaptive MAC (LDTA-MAC) in which they propose a dynamic slot allocation of Guaranteed Time Slots (GTS) in the CFP to accommodate traffic variations. The superframe consists of a beacon, a CAP with fixed duration, a CFP used to transmit time critical data and an IP. The boarder line between CFP and IP is variable based on the number of GTS requests. When no GTS requests are encountered, the superframe will solely consist of the beacon and the CAP; and when there are a lot of GTS requests, the CFP duration can be extended till the end of the superframe. Even though this protocol aims to increase the number of time slots needed for time critical data, the CFP duration is incremental with GTS demand; and since many nodes will send their critical data simultaneously upon emergency, then either the superframe duration should be long enough to acquire needed slots, or packets will not find available slots. In both cases, high transmission delay will be induced, which is not feasible for emergency data.

A traffic Priority and Load Adaptive MAC (PLA-MAC) is presented in [13]. The corresponding superframe is similar to that of PNP-MAC, but the CFP duration is dynamic and can be extended till the end of the superframe like in LDTA-MAC. PLA-MAC sends data by priority, and emergency data are given the highest priority through allocating ETS used to transmit urgent data before DTS in the superframe. Even though emergency data are given the highest priority, this protocol may not guarantee immediate handling of critical data as ETS interval increases significantly with the number of nodes carrying emergency data which is not viable during emergency. This is in addition to the fact that some nodes may not succeed to send request for CFP slot during CAP due to the high contention that occurs in heavy traffic.

The authors in [14] propose a Context Aware MAC (CA-MAC) in which the duty cycle and sampling rate of the nodes depends on the context variation of BSN. When the coordinator detects a change in the context through data analysis and processing, it triggers an emergency state during which nodes sensing relevant data increase their duty cycle and sampling rate, and will be therefore allocated more slots for data transmission; whereas the other nodes can either decrease their sampling rate or refrain from transmitting data. This protocol guarantees reliable transmission of critical data through increasing channel availability; however, it does not ensure their transmission time within the strict delay requirements which is crucial in emergencies.

In [15], a Contention over Reservation MAC (CoR-MAC) is proposed to decrease the transmission delay of critical data. In this protocol, the corresponding superframe consists of a beacon, CFP, CAP, and IP. Nodes that might generate emergency data are allocated dedicated time slots in CFP, and the CAP duration shrinks as CFP expands. To increase channel use, these slots can be used by other nodes when they are not needed. The protocol thus operates as follows: emergency data are transmitted over both the CAP and CFP; time critical data are transmitted over CAP or reserved slots in the CFP if they are not used by emergency data, and non time critical data are transmitted over CAP or CFP as long as they are not used by both emergency and critical data. Even though this algorithm increases the channel use, it does not guarantee timely delivery of urgent data because the CFP duration might become very long when the number of nodes holding emergency data increases. Also, narrowing CAP to expand CFP will increase the congestion in the CAP which will further increment the transmission delay.

In [16], the authors propose a scheme that increases the probability of urgent data transmission through setting a maximum number of transmission retry for both urgent and regular data. This protocol guarantees reliable delivery of emergency data, but does not consider the corresponding transmission delay.

The authors in [17] propose a Priority Guaranteed MAC (PG-MAC) in which traffic is divided into two classes: medical or urgent traffic and Consumer Electronics (CE). The corresponding superframe is formed of both CAP and CFP. CAP is used for reservation requests, and CFP slots are used for data transmission. Both CAP and CFP are divided into two parts, one for every data class type. This will guarantee channel availability for urgent data even when CE traffic is heavy. However, this protocol does not ensure immediate delivery of urgent data, even when CFP slots for urgent traffic are no longer available and CE slots are empty.

Other MAC protocols suggested in literature such as [19,20,21] propose adaptive wake-up interval mechanisms for efficient transmission of variable traffic. For instance, the authors in [19] propose a Traffic-adaptive MAC (TaMAC) in which they assign a traffic-based wake-up mechanism for normal traffic, and a wake-up radio mechanism for emergency or on-demand traffic. In the traffic-based wake-up mechanism, the traffic patterns of nodes holding normal data are arranged in a table, and thus, the coordinator wakes-up the nodes only at the right time, otherwise they stay in sleep mode. Whereas in the wake-up radio mechanism, nodes holding emergency data send wake-up signal to the coordinator whenever they have data to send. Even though this protocol aims to decrease nodes’ idle listening and overhearing, it does not consider the simultaneous traffic burst from many sensors during emergency situations, which increases the contention between emergency nodes for channel access; and thus this protocol does not ensure fast delivery of urgent data. In [20], authors proposed a Traffic-Aware Dynamic MAC Protocol (TAD-MAC) for both invasive and non-invasive WBAN. In this protocol, every node dynamically adapts its wake-up interval based on its traffic information obtained from the Traffic Status Register (TSR) bank. The proposed algorithm keeps updating the node’s wake-up interval until converging to a steady state in which idle listening is minimal. Also, in [21], authors developed a Heuristic Self-adaptive MAC (HS-MAC) for resource constrained WBAN systems, to ensure that TAD-MAC protocol always converges toward a steady state value and reaches minimal idle listening, through re-configuring the wake-up schedules of the nodes. Both protocols (TAD-MAC and HS-MAC) perform well under normal traffic, but they do no consider the behavior of emergency traffic where many high traffic nodes will need to send their critical data simultaneously. The traffic burst increases the wake-up interval of the emergency nodes. and therefore, multiple nodes carrying urgent data will be active at the same time, which increases the contention for channel access. Thus, these schemes do not guarantee transmission of urgent data with minimal delay.

The above discussion shows that the existing MAC protocols do not guarantee timely delivery of emergency data due to the inability of DTMA mechanism used in the CFP to handle heavy traffic, and the contention nature of CSMA/CA mechanism adopted in the CAP. Also, the continuous medium listening in the CAP decreases the energy efficiency of these protocols even under low traffic [22]. For this reason, we propose a flexible and efficient emergency aware hybrid DTDMA/DS-CDMA MAC protocol able to answer the BSN requirements in transmitting emergency data within strict limits, while maintaining high energy efficiency during periodic observation.

## 3. Hybrid DTDMA/DS-CDMA Protocol

We consider that sensors are distributed around a coordinator node in star topology architecture. In normal conditions (non-emergency cases), nodes send their data to the coordinator node at constant packet rate that ranges between 1 and 20 packets/s. When the coordinator node identifies abnormalities in physiological signal by processing and analyzing received data, the emergency state is triggered, and the coordinator requests from sensor nodes to send more data to carry out precise recognition; thus nodes will increase their sampling rate which will significantly increase the corresponding traffic rate (up to 100 packet/s) [6].

The proposed scheme is a hybrid DTDMA/DS-CDMA protocol that combines the advantages of DTDMA and DS-CDMA, in order to adapt to BSN traffic variations and different data requirements.

### 3.1. DTDMA Protocol

Dynamic Time Division Multiple Access (DTDMA) is a contention-free scheduled protocol in which nodes transmits their data one after another in dedicated time slots. Variable number of time slots are dynamically allocated to different nodes based on the corresponding traffic rate. DTDMA induces a low delay in periodic traffic; however, the delay remarkably increases in high traffic environment due to un-avoided queuing. The main advantage of DTDMA remains its high energy efficiency since nodes only transmit data in their allocated time slots, and remain inactive all the other times. They do not encounter collisions, overhearing or idle listening problems [23,24].

### 3.2. DS-CDMA

In Direct Sequence Code Division Multiple Access (DS-CDMA), nodes are assigned different codes, and can therefore send their data simultaneously over the same frequency. The node’s assigned code is multiplied by the node’s original data to produce its encoded data, i.e., encoded data = (original data) × (assigned code). This is why it is called Direct Sequence-CDMA or DS-CDMA. The spreading codes are computed using orthogonal Walsh-Hadamard codes since it is one of the best orthogonal code generation techniques used to mitigate Multiple Access Interference (MAI) problem that arises from the asynchronous nature of BSN [25,26]. In general, the energy consumed by nodes for data processing prior to transmission includes compression, encoding, and encryption of data; and is modeled as “a constant per packet energy plus a term that is directly proportional to the length of the packet” [27]. Therefore, the computation needed to encode the packets increases the node’s energy consumption. However, the main advantage of DS-CDMA is that nodes can transmit their data simultaneously over the channel, which leads to low induced delays even in high traffic environments [18].

### 3.3. Proposed DTDMA/DS-CDMA MAC Protocol

The proposed hybrid DTDMA/DS-CDMA scheme works as follows: During regular monitoring, the traffic is periodic and relatively low. For this reason, nodes send their data in their allocated time slots using DTDMA mechanism. DTDMA is chosen since it has high delay and energy efficiency in periodic traffic environment. When emergency occurs, many nodes will need to transmit their critical data simultaneously to the coordinator node within strict delay requirements; accordingly, the proposed scheme dynamically allocates emergency slots that accept concurrent data from multiple sensors through assigning them different codes using DS-CDMA mechanism. The emergency data are therefore transmitted simultaneously over the same time slots with the lowest delay, as DS-CDMA has high delay efficiency in heavy traffic [18]. The other nodes holding non-critical data will continue transmitting their data in the remaining time slots one after another via DTMA mechanism. Therefore, only nodes holding critical data will need to be assigned different spreading codes, which provides a balance between energy and delay efficiency.

Therefore proposed hybrid DTDMA/DS-CDMA MAC protocol has three main advantages: first it is adaptive as it dynamically allocates slots based on traffic requirements and can therefore adapt to BSN traffic variations; second it is emergency aware as it handles emergency data efficiently by guaranteeing their delivery at minimal delays while trying to balance energy consumption, and third it works well in non-emergency cases as it guarantees good delay and energy efficiency.

#### 3.3.1. Proposed Superframe Structure

**Figure 1:**
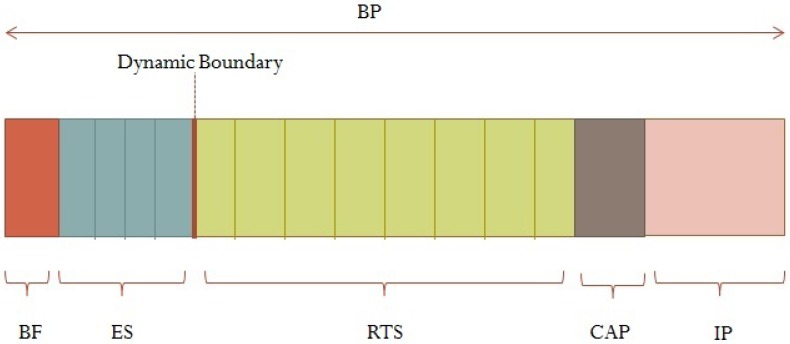
Proposed MAC Superframe Structure.

The superframe of the proposed scheme is presented in Figure 1. It is formed of a Beacon Field (BF), Emergency Slots (ES), Regular Traffic Slots (RTS), an optional Contention Access Period (CAP), and an Inactive Period (IP). The Beacon Period (BP) is the period of the superframe.

The BF consists of a beacon packet sent by the coordinator to ensure network synchronization and to advertise information like slot allocation, spreading codes, length of BP, number of slots in ES and RTS periods, as well as CAP and IP lengths.

The ES are used for emergency data transmission. These slots are able to accommodate data from multiple nodes through assigning them different codes using DS-CDMA mechanism. The boundary of the ES period is dynamic and depends on the highest packet rate of the generated emergency data. For instance, in non-emergency cases, no ES slots will be allocated, and the superframe will be solely formed of BP, RTS, optional CAP and IP; whereas when emergency occurs, ES period increases to accommodate the maximum urgent data rate. However, the ES period has a maximum limit of 120 ms to guarantee that critical data are delivered within their corresponding delay requirements (less than 125 ms) [6].

The RTS are used to transmit regular periodic traffic, where nodes send their data each in its dynamically allocated time slot using DTDMA mechanism.

The CAP period is optional, and is used to allow new nodes to join the network. It uses CSMA/CA scheme to access the channel. The CAP period is basically used by non-medical sensors, or by temporal medical sensor nodes that join the network in specific medical conditions.

The IP is used to save energy as nodes enter into sleep mode, and is configurable, based on the ES and RTS duration. For instance, the IP increases in non-emergency cases when the traffic is relatively low and decreases when the traffic is high.

#### 3.3.2. Slot Allocation Procedure

**Figure 2:**
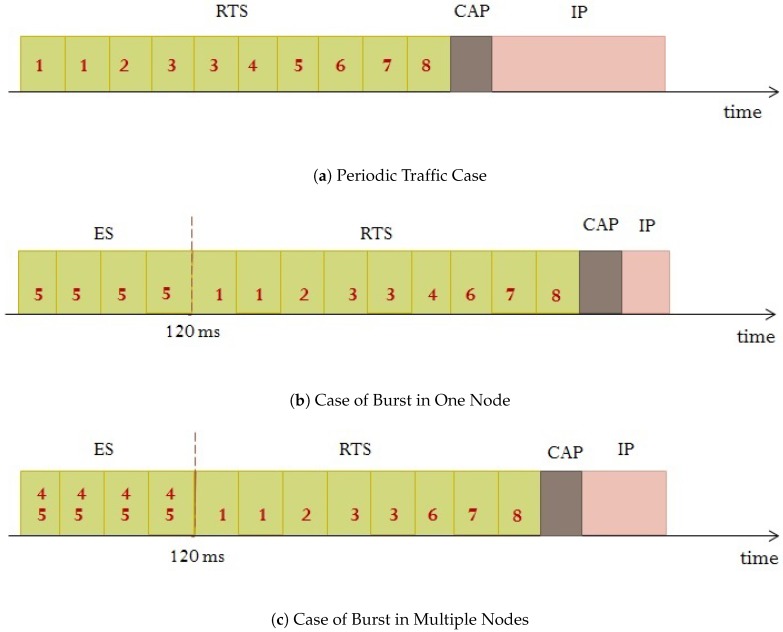
Slot Allocation Procedure.

The slot allocation procedure is illustrated in Figure 2. Consider a BSN formed of eight sensors distributed over the body of an athlete to monitor his health condition. Figure 2a presents slot allocation during normal conditions such as training. In this case, ES period does not exist and every node sends its data in its dynamically allocated time slots in the RTS period.

The optimal number of slots that should be allocated for every node is:

(1) $$\begin{array}{c} Slots=\frac{PR\cdot BP}{NP} \end{array}$$

where:

$$\begin{array}{c} PR:\mathrm{Packet}\;\mathrm{Rate}\\ BP:\mathrm{Beacon}\;\mathrm{Period}\\ NP:\mathrm{Number}\;\mathrm{of}\;\mathrm{packets}\;\mathrm{in}\;a\;\mathrm{slot} \end{array}$$

and

(2) $$\begin{array}{c} NP=\frac{\mathrm{Slot}\;\mathrm{Duration}}{\mathrm{Packet}\;\mathrm{Duration}} \end{array}$$

Equation (1) provides an efficient slot allocation mechanism since it helps nodes achieving the highest throughput, and assigning more slots will not enhance the performance [28]. This equation shows that the number of allocated slots is directly proportional to the packet rate. For example, if the channel rate is 250 Kbps, and the payload size is 32 bytes, then every packet needs 1.024 ms to be transmitted via BSN radio. The total packet duration equals radio transmission time + ACK + TX, where TX is the radio transition time needed to activate transmission mode. ACK and TX depends on the transceiver used; they are equal to 0.16 ms in Nordic nRF24L01 that is widely used in BSNs [29]. Thus, the total duration for a packet 1.184 ms. Assuming a slot duration of 5 ms, then every slot can accommodate 4 packets. Nodes in regular observation have traffic rates ranging between 1–20 packets/s; therefore for a BP of 240 ms, the number of slots that should be allocated to nodes with traffic rate of 20 packets/s is 2, whereas only 1 slot should be allocated to nodes a rate of 5 packets/s.

When a sudden change in the person’s activity or environment occurs, like for example if the monitored athlete’s heartbeat increases above a certain threshold, the ECG sensor holding information about heart rate (node 5 in this example) experiences a burst of traffic due to emergency; in this case, the coordinator reserves ES for this node at the beginning of the superframe before RTS to guarantee fast delivery of urgent data as shown in Figure 2b. The boundary of the ES is based on the traffic rate of the emergency data. Equation (1) is used to compute the needed number of slots. However, the ES period duration cannot exceed 120 ms in order to satisfy the emergency delay requirements, and therefore, the maximum number of slots that can be assigned in the ES period is equal to 120 ms divided by the slot duration. For instance, if the slot duration is 5 ms, then the number of slots in the ES period should be less or equal to 24 slots, and the ES boundary will then vary from zero slot when no emergency data is encountered to a maximum of 24 slots based on the emergency data traffic rate. The other nodes will continue transmitting their periodic data in the RTS period using DTDMA in the aim of balancing the energy consumption.

In many emergency cases, multiple nodes need to send urgent data to the coordinator, due to high traffic correlation. For example, if the athlete faints, both blood pressure and ECG sensors (nodes 4 and 5 in this example) enter into emergency state and experience burst of traffic. In such case, ES will be used to transmit data from these sensors simultaneously by assigning them different codes as illustrated in Figure 2c. The duration of ES is based on the maximum generated traffic rate. Following this example, if node 4 traffic rate is 70 packets/s, and that of node 5 is 100 packets/s, then the ES duration will be configured to accommodate the traffic rate of node 5, and the ES period will have the duration of 6 slots that is 30 ms.

The dynamic allocation procedure for nodes carrying emergency data is shown in Algorithm 1. If only one node has urgent data to send, the coordinator computes the number of needed slots based on the PR of this node using Equation (1). If more than one node carry urgent data, the coordinator assigns different spreading codes to every node, and computes the number of needed slots based on the maximum received PR using Equation (1). In both cases, the algorithm ensures that the the number of allocated slots do not exceed the maximum number of slots allowed in the ES period to guarantee that critical data is delivered within strict delay requirements (120 ms). The coordinator then allocates the slots in the ES period, and updates the spreading code and slot allocation information in the beacon frame.

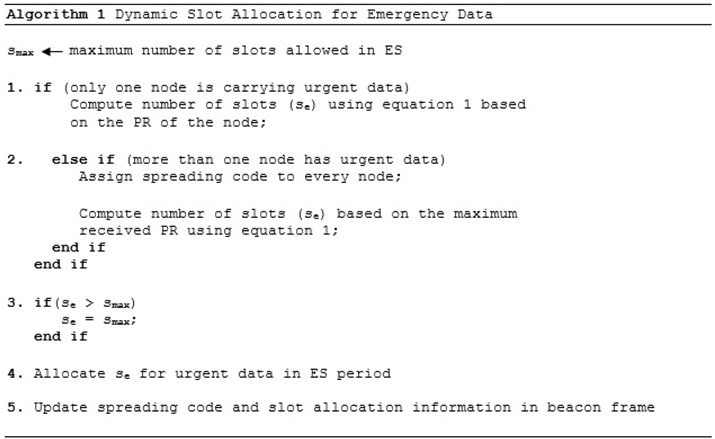



The dynamic allocation procedure for nodes carrying regular data is shown in Algorithm 2. As explained in Algorithm 1, the coordinator assigns spreading codes to emergency nodes. Therefore when a node is no longer carrying emergency data, the coordinator frees it from the spreading code (it will no longer send spreading code to this node). The coordinator computes the number of needed slots for every node based on the corresponding PR using Equation (1); it then allocates these slots in the RTS period, and updates the spreading code and allocation information in the beacon frame.

#### 3.3.3. Protocol Data Transfer Operation

**Figure 3:**
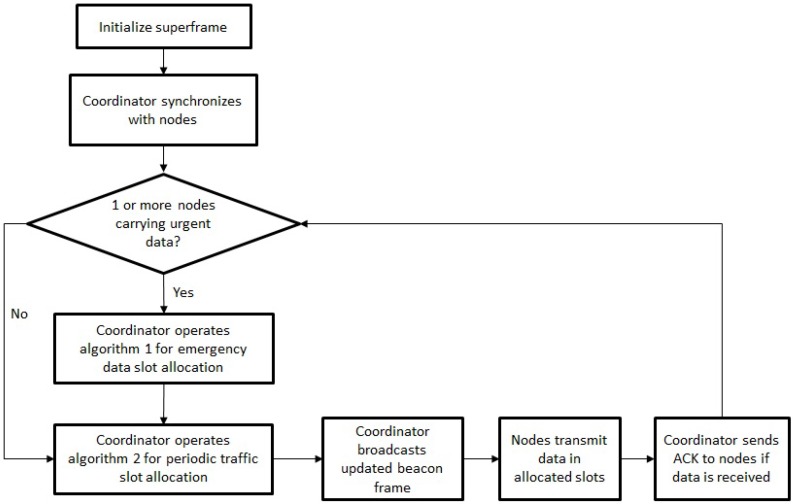
Block Diagram of Protocol Data Transfer Operation.

The protocol’s data transfer procedure is presented in Figure 3.

The coordinator initializes the superframe and synchronizes with existing nodes through broadcasting a beacon frame including the coordinator’s ID and the superframe structure. In non-emergency cases, all nodes carry regular data; therefore, the coordinator operates Algorithm 2 to allocate appropriate slots in the RTS period. However, if emergency is triggered, one or more nodes will have emergency data to send, the coordinator will then operate Algorithm 1 to dynamically allocate slots to nodes with emergency data in the ES period; it then operates Algorithm 2 to dynamically allocate slots to remaining nodes in the RTS period. After slot allocation, the coordinator broadcasts the updated beacon frame, allowing nodes to send their data each in its allocated slots. The coordinator then sends ACK to nodes if data is received.

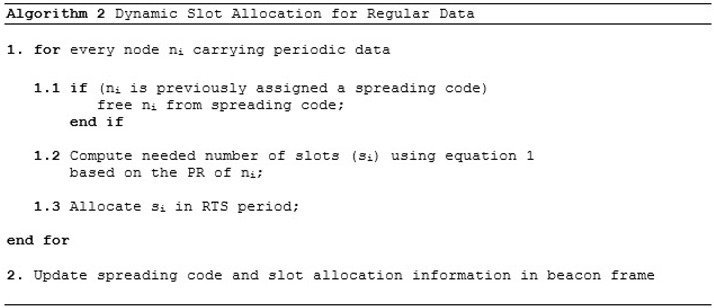



## 4. Evaluation of the Proposed Scheme

To evaluate the performance of the proposed hybrid DTDMA/DS-CDMA MAC protocol, the suggested scheme is compared to DTMA, DS-CDMA, as well as PLA-MAC [13] and CoR-MAC [15] hybrid MACs. PLA-MAC is chosen since it is a traffic adaptive protocol that gives priority to emergency data and uses the concept of dynamic CFP configuration based on traffic demand, which is close to the concept of the proposed scheme; and CoR-MAC is chosen since it is a recent protocol for urgent data transmission that allows slots sharing by multiple sensors which is also close to the proposed scheme. Delay, packet drop percentage, and energy consumption of the three schemes are simulated, and the results are discussed to compare the performance of the proposed protocol to other existing mechanisms.

### 4.1. Performance Metrics

The following three performance matrices were used to assess the performance of the proposed MAC:

- *Average Packet Delay*: it is the interval of time between the generation of a packet by the sensor node, to the moment when it is successfully received by the coordinator. This metric shows the ability of the MAC protocol to deliver packets with minimal delay.
- *Percentage of Dropped Packets*: it is the ratio of the number of packets that are dropped due to failed transmission to the total number of generated packets by the nodes. This metric allows to evaluate the reliability of the compared schemes and their ability to transmit packets successfully.
- *Energy Consumption*: it represents the percentage of energy consumed by each protocol with respect to the total energy consumed by the system to send the data. this metric is used to assess the energy efficiency o the compared protocols.

### 4.2. Simulation Parameters

We consider a BSN formed of 16 sensor nodes distributed over the human body in a star topology architecture around the coordinator that is placed on the center of the body. Periodic (regular) traffic uses Constant Bit Rate (CBR) traffic model, and the traffic rate of nodes is set between 1 and 20 packets/s to reflect periodic low to moderate traffic; whereas emergency traffic uses Poisson model to emulate random packet arrival, and the traffic rate of the emergency data is set to 100 packets/s to approximate very high traffic [6,8]. As stated earlier, when emergency is triggered, like for example when a monitored patient with heart attack blackouts, sensors holding critical data such as heart rate (ECG) should be given the highest importance to guarantee instant and reliable delivery of information, while other data like EMG, motion, and visual sensors data become less important [8]. In addition, since traffic in BSN is highly correlated, many other sensors will also have critical data to send, like blood pressure, oxygen sensors in our example. For this reason, Simulations were performed for different scenarios: non-emergency scenario in which all nodes carried regular traffic (number of nodes with urgent data = 0), and various emergency scenarios in which different number of nodes (1 to 8 nodes) carried emergency data. The aim of this simulation is to study the behavior of the three MAC protocols when multiple number of nodes request simultaneous transmission of critical data to the coordinator node due to traffic correlation. During all the above simulations, the payload size is assumed to be 32 bytes.

Also, to study the impact of varying payload size, Simulations were repeated for payload sizes of 10, 30, 50, and 70 bytes, and the behavior of the proposed scheme is assessed when 25% of nodes (4 nodes) are carrying emergency data.

The performance of the three listed protocols is assessed using OPNET simulator. The simulation parameters are summarized in Table 1.

### 4.3. Simulation Results and Discussion

#### 4.3.1. Performance for Varying Number of Emergency Nodes

**Figure 4:**
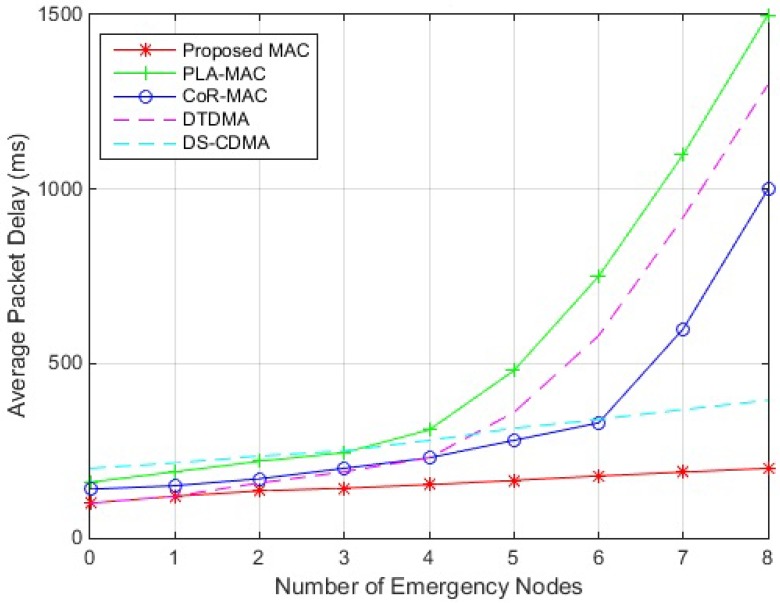
Delay Performance of MAC Protocols.

The average packet delay of the proposed hybrid DTMA/DS-CDMA scheme, PLA-MAC, CoR-MAC, as well as DTDMA and DS-CDMA, are presented in Figure 4. Results show that the proposed scheme outperforms the others and induces the lowest packet delay during periodic observation (number of emergency nodes = 0) and for different number of nodes delivering emergency data. The reason is that the proposed scheme sends urgent data simultaneously over the same period (ES period), which guarantees their immediate delivery without further delay even when the number of nodes holding emergency data increases; this simultaneous transmission of data will also decrease the number of slots allocated in the RTS period, which will in return decreases the delay of regular traffic packets, and results in low average packet delay in the network. The delay induced by the proposed scheme slightly increases with the number of nodes holding emergency data since spreading codes should be assigned to more nodes. PLA-MAC induces the highest delay among the compared schemes since it assigns GTS slots in the ETS period based on traffic demands, and therefore as the number of nodes holding emergency data increases, the ETS duration expands, so the transmission delays of both emergency and regular packets will increase. Also, nodes use CSMA/CA mechanism in CAP to reserve their time slots which will further increase the delay since nodes have to always listen to the medium to make sure that it is free before transmitting their requests. The PLA-MAC delay significantly increases when the number of nodes generating emergency data becomes high (more than 4 nodes in our simulation); this is because the superframe becomes saturated and cannot accommodate more time slots; requests for slot reservation will be therefore declined, and nodes will have to wait for a long time before a time slot is available, which will drastically increase the packets delay. CoR-MAC performs better than PLA-MAC, and can handle higher number of emergency nodes before superframe saturation since slots allocated for emergency traffic can be used by nodes holding regular data traffic, taking advantage of the sporadic nature of emergency traffic. However, CoR-MAC under performs the proposed scheme even when the superframe is not saturated since the CFP duration in CoR-MAC increases with the number of nodes holding emergency data, whereas all nodes holding urgent data are served simultaneously at the beginning of the superframe in the proposed scheme. Moreover, when the number of nodes holding urgent data increases above a certain threshold (6 nodes in this case), the superframe saturates and the delay of CoR-MAC increases intensely. Concerning DTDMA protocol, the induced delay is similar to that of the proposed scheme in non-emergency cases and when only one node sends emergency data since both scheme behave the same way. However, DTDMA becomes less delay efficient than the proposed scheme when the number of nodes carrying emergency data increases, since more slot will be assigned leading to higher CFP duration; the delay becomes unmanageable in DTDMA when the number of nodes carrying emergency data becomes high (5 nodes in the simulation) due to superframe saturation. DTDMA performs slightly better than CoR-MAC before superframe saturation since nodes do not compete to reserve time slots; however, DTDMA superframe saturates faster than CoR-MAC since unlike DTDMA, CoR-MAC allows several nodes to share the same slot. The above analysis indicates that PLA-MAC, CoR-MAC and DTDMA fail in handling the traffic correlation property of BSN. As for DS-CDMA, codes are assigned and should be encoded to all packets regardless of the nodes traffic type. This makes it less delay efficient than he other protocols in non-emergency cases when traffic is periodic and low. The delay induced by DS-CDMA increases with the number of nodes carrying emergency data as codes should be assigned to more packets; however, the delay remains manageable even when the number of nodes carrying emergency data becomes high due to high channel availability.

**Figure 5:**
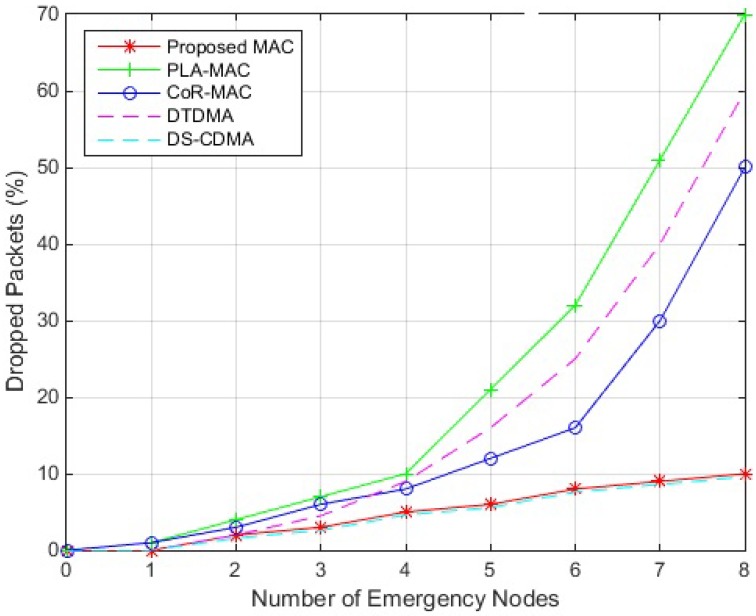
Packet Drop Performance of MAC Protocols.

The percentage of dropped packets simulation results are illustrated in Figure 5. They show that the proposed scheme and DS-CDMA have close performance and are the most reliable among the compared protocols, as they lead to the lowest packet drops. They particularly outperform the others when the number of nodes holding urgent data becomes high, since in DS-CDMA, all nodes can transmit their data simultaneously over the channel regardless to the traffic type; and in the proposed scheme, urgent data is transmitted simultaneously at the beginning of the superframe, allowing other nodes holding regular traffic data to find available slots to transmit their data in the RTS due to the low congestion in this period. The probability of successful transmission of both emergency and regular packets will therefore increase for both protocols due to high channel availability. PLA-MAC has the highest packet drop percentage that significantly increases when the number of nodes holding emergency data becomes high, since requests for slot allocation will be declined due to the limited number of time slots. CoR-MAC performs better than PLA-MAC since slots allocated for emergency data can be used by regular traffic data which will reduce the contention in the CAP, and will increase the probability of finding slots during CFP. DTDMA protocol outperforms PLA-MAC and CoR-MAC when the number of emergency nodes is low due to absence of contention for slot reservation; however, the percentage of dropped packets largely increases when the number of emergency nodes become high due to superframe saturation.

**Figure 6:**
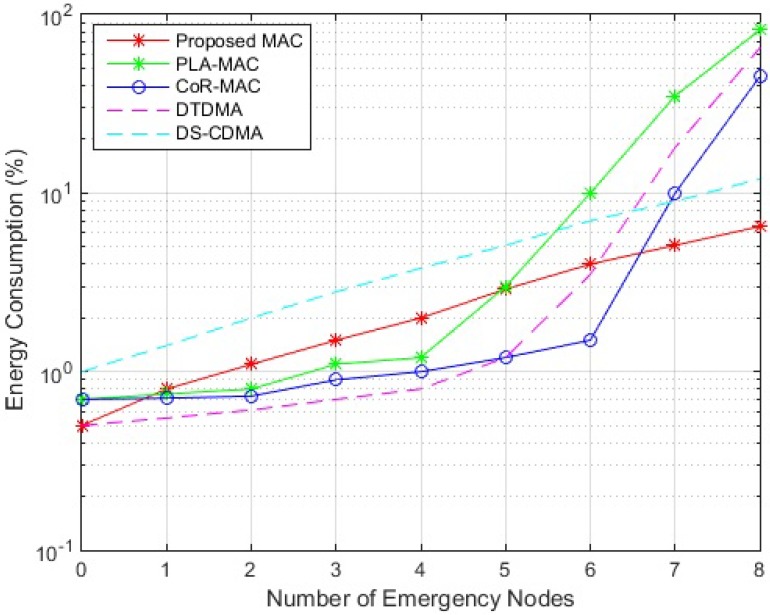
Energy Performance of MAC Protocols.

Figure 6 presents the energy consumption performance of the three compared protocols. Results show that in non-emergency cases, the proposed scheme and DTDMA are the most energy efficient, since the proposed scheme follows DTDMA operation where nodes sends their data in their time slots only and remains inactive all the other times. PLA-MAC and CoR-MAC are less energy efficient than the proposed scheme during periodic observation, since they both use CAP based on CSMA/CA for GTS allocation, which will actuate higher power consumption resulting from CSMA/CA continuous collision detection and avoidance requirements. DS-CDMA consumes the highest energy among the compared schemes since spreading codes are encoded to every packet of nodes, with leads to high data processing energy. In emergency cases, and as the number of nodes holding emergency data increases, the energy consumption of the proposed scheme increases since data processing energy needed to encode packets generated from multiple sensors increments. However, the main requirement in BSN is to deliver emergency data immediately and with the lowest packet drop rate. Therefore the computation requirements of can be traded with its low delay and packet loss characteristics. Also, in the proposed scheme, the regular traffic data will still be transmitted over RTS period via DTMA mechanism, which will balance the energy consumption; i.e., only urgent packets will be assigned spreading codes. Similar to the proposed protocol, DS-CDMA energy consumption increases with the number of nodes carrying emergency data due to higher data processing requirements; however, DS-CDMA remains less energy efficient than the proposed scheme since all packets of all nodes sould be encoded which leads to higher energy consumption. As for DTDMA, PLA-MAC and CoR-MAC, their consumed energy slightly increases when more nodes generate emergency traffic, since more slots will be assigned to nodes, forcing them to be awake for longer times. However, the energy consumption of DTDMA, PLA-MAC and CoR-MAC increases significantly when their superframe saturates, as nodes will remain active as long as they have packets, and keep re-transmitting reservation requests until finding available time slot which will increase the energy consumption. Therefore, these three protocols are not energy efficient as the probability that multiple nodes send their data simultaneously to the coordinator is very high in emergency situations.

The delay, packet drop and energy consumption simulations showed that the proposed scheme outperforms other existing protocols and offers a flexible and efficient way to adapt to BSN dynamic traffic nature.

#### 4.3.2. Performance for Varying Payload Size

**Figure 7:**
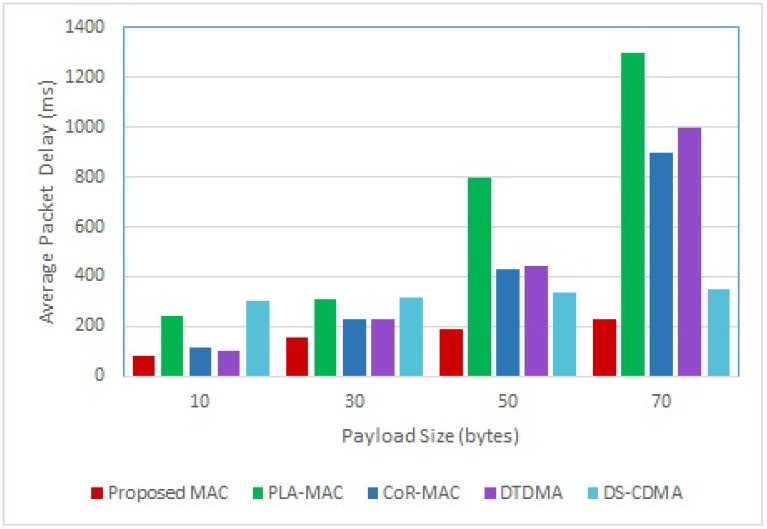
Delay Performance for Varying Payload Size.

The delay performance of the five compared schemes for varying payload size is illustrated in Figure 7. In the simulation, we consider that four nodes carry emergency data. Results show that the average packet delay increases with the payload size for all compared schemes, and the proposed scheme is the most delay efficient for various payload sizes. For instance, more processing time is required to encode large packets for all nodes in DS-CDMA, and more GTS slots should be assigned to nodes in DTDMA, PLA-MAC and CoR-MAC since fewer packets can be transmitted in one slot. The delay of the last three schemes significantly increases when the payload size is large (50 bytes for PLA-MAc and 70 bytes for CoR-MAC and DTDMA), as the corresponding superframes saturates and nodes will have to wait for slots to become available. As for the proposed scheme, unlike DS-CDMA, packets from only four nodes should be encoded, and unlike DTDMA, PLA-MAC and CoR-MAC, even though more slots should be assigned in the ES and RTS period to accommodate large packets, emergency packets are sent simultaneously over the same ES period, which increases the scheme delay efficiency.

**Figure 8:**
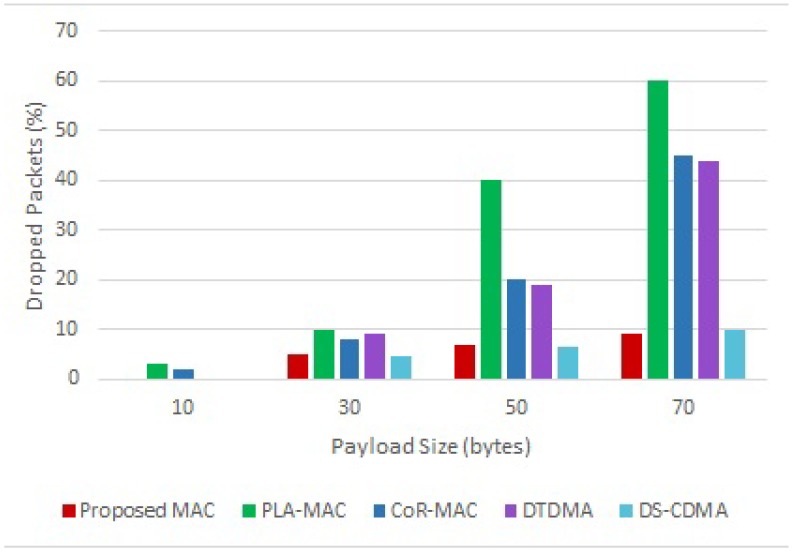
Packet Drop for Varying Payload Size.

The packet drop percentage results for varying payload size are presented in Figure 8. Results show that the percentage of dropped packets increases with the increase of the payload size. The proposed scheme as well as DS-CDMA exhibit the lowest packet drop percentage among the compared protocols for different payload sizes due to high channel availability that ensures the delivery of large packets. Whereas in DTDMA, PLA-MAC, and CoR-MAC, large packets will need to occupy more slots, and therefore many packets will be dropped due to limitted number of available slots.

**Figure 9:**
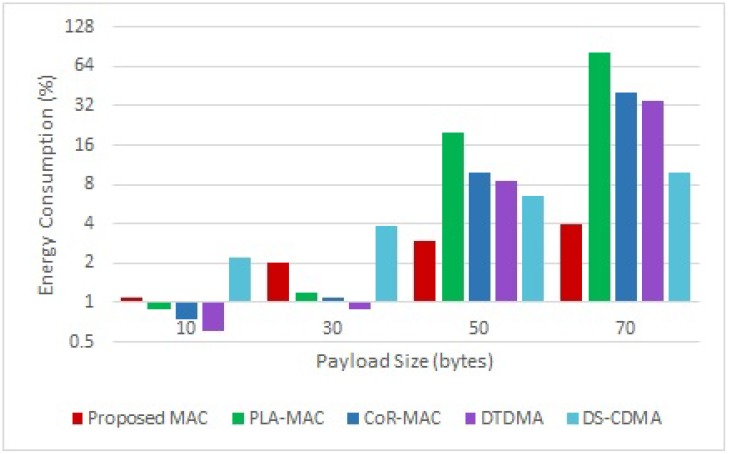
Energy Consumption for Varying Payload Size.

The packet energy consumption results for varying payload size are presented in Figure 9. Results show that DTDMA, LDA-MAC and CoR-MAC are more energy efficient than DS-CDMA and the proposed scheme when the payload size is small, since nodes in DTDMA, LDA-MAC, and CoR-MAC are only active in their dedicated slots, and the number of assigned slots per node is low when the payload size is small. Whereas the proposed scheme and DS-CDMA consume more energy due to data processing required in packets encoding. However, when the payload size increases, the proposed scheme and DS-CDMA become more energy efficient than DTDMA, LDA-MAC and CoR-MAC, as the superfarme structure of these three protocols saturates quickly and therefore nodes will stay active until finding an available slot which will significantly increase the corresponding energy consumption. Also, the proposed scheme outperforms DS-CDMA in terms of energy consumption, since data processing is only performed on four nodes, and nodes carrying regular traffic are only active in their slots.

The above results show that the proposed scheme outperforms other protocols in terms of delay and packet drop percentage under varying payload size. It also outperforms the other schemes in terms of energy consumption for large payload sizes.

## 5. Conclusions and Future Work

In this paper, a new efficient emergency aware hybrid DTDMA/DS-CDMA MAC protocol is proposed to guarantee fast transmission of data in emergency cases, while maintaining high energy efficiency during periodic observation. The proposed scheme takes advantage of the low delay induced by DS-CDMA in high traffic, and the low energy consumed by DTDMA in periodic traffic. The suggested protocol was compared to other existing schemes with respect to delay, packet drop and energy consumption for various number of nodes carrying urgent data. Results showed that it outperforms the others as it is able to achieve the lowest delay and packet drop percentage even when multiple sensors request simultaneous transmission of emergency data; it also offers the highest energy efficiency during periodic observation, while adjusting the energy consumption during emergency by assigning spreading codes only to nodes holding emergency data. The impact of varying payload size was then analyzed, and results also showed that the proposed scheme achieves the lowest delay and packet drop percentage among the compared schemes for different payload sizes, it is also the most energy efficient among others for large payload sizes. Future work includes studying the behavior of the proposed scheme with respect to other metrics like throughput and scalability, as well as addressing other BSN challenges such as channel fading. Also, the proposed MAC protocol is analyzed for single body monitoring; this analysis can therefore be extended to study the performance of the proposed scheme in Collaborative BSN (CBSN) applications.

## Figures and Tables

**Table 1:** SIMULATION PARAMETERS.

Parameter	Value
Number of Nodes	16
Topology Size	2 m × 1 m
Topology Type	Star
Number of Nodes with Urgent Data	0–8
Packet Rate for Regular Traffic	1–20 packets/s
Packet Rate for Emergency Traffic	100 packets/s
Regular Traffic Model	CBR
Emergency Traffic Model	Poisson
Payload Size	10, 30, 50, 70 Bytes
Slot Duration	5 ms
Channel Rate	250 Kbps
